# Reactive Oxygen Species in Skeletal Muscle Signaling

**DOI:** 10.1155/2012/982794

**Published:** 2011-12-05

**Authors:** Elena Barbieri, Piero Sestili

**Affiliations:** Dipartimento di Scienze Biomolecolari, Università degli Studi di Urbino “Carlo Bo”, via Saffi 2, 61029 Urbino, Italy

## Abstract

Generation of reactive oxygen species (ROS) is a ubiquitous phenomenon in eukaryotic cells' life. Up to the 1990s of the past century, ROS have been solely considered as toxic species resulting in oxidative stress, pathogenesis and aging. However, there is now clear evidence that ROS are not merely toxic species but also—within certain concentrations—useful signaling molecules regulating physiological processes. During intense skeletal muscle contractile activity myotubes' mitochondria generate high ROS flows: this renders skeletal muscle a tissue where ROS hold a particular relevance. According to their hormetic nature, in muscles ROS may trigger different signaling pathways leading to diverging responses, from adaptation to cell death. Whether a “positive” or “negative” response will prevail depends on many variables such as, among others, the site of ROS production, the persistence of ROS flow or target cells' antioxidant status. In this light, a specific threshold of physiological ROS concentrations above which ROS exert negative, toxic effects is hard to determine, and the concept of “physiologically compatible” levels of ROS would better fit with such a dynamic scenario. In this review these concepts will be discussed along with the most relevant signaling pathways triggered and/or affected by ROS in skeletal muscle.

## 1. Introduction

**Figure 1:**
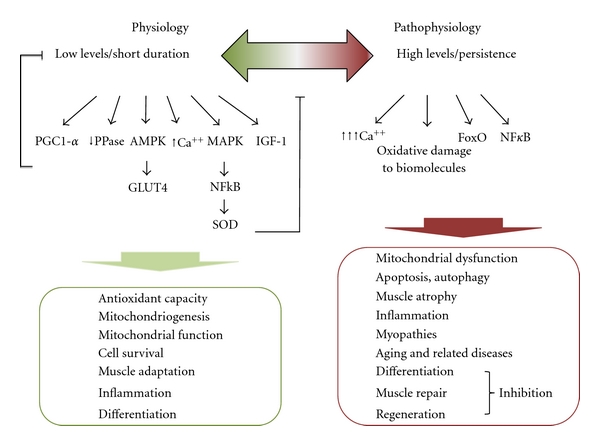
Major signaling pathways triggered and/or affected by ROS in skeletal muscle. Low levels of ROS activate specific key signaling molecules such as PGC-1*α*, AMPK, and MAPK, which control cellular mechanisms for muscle adaptation (oxidative metabolism, mitochondrial biogenesis, and mitochondrial functionality) as well as antioxidant enzymes that function as backregulators of intracellular ROS levels. Slight ROS accumulation also inhibits PPases and promotes the phosphorylation state of many proteins involved in the muscle signaling responses. Moreover, low levels of ROS play an important role in inducing upregulation of growth factors such as IGF-1, which has beneficial effects in muscle protein balance, supports oxidative metabolism, and contributes to the development of an oxidant-resistant phenotype, therefore preventing oxidative damage and chronic diseases. Thus, low levels of ROS elicit positive effects on physiological muscle responses. By contrast high levels of ROS cause functional oxidative damages of proteins, lipids, nucleic acids and cell components, induce a significant rise of intracellular [Ca^2+^], and promote signaling cascades for apoptosis or autophagy *via* NF-*κ*B or FoxO paths. For these reasons high ROS levels are reputed to act as etiological, or at least exacerbating factors in muscle atrophy, sarcopenia, wasting, and chronic-/aging-related muscle diseases and myopathies. Depending on their level/persistence, ROS may also turn the same process from “physiologic” into “pathologic”, as in the case of inflammation.

Oxidative stressors, such as reactive oxygen species (ROS), have been initially and long considered as merely deleterious species to skeletal muscle tissue. Indeed, since the 1980s abundant evidence clearly indicated that ROS play a pathogenic role in inherited muscular dystrophies [1] and have then been identified as concausal factors in various muscular diseases [2–5]. However, and thereafter, accumulating evidence indicated that ROS, at least within concentrations emerging from physiological conditions, could also play a positive role in physiologically relevant processes in muscle cells. As an example, inflammation-derived ROS play a contradictory role in muscle repair [2]: in combination with other actors such as growth factors and chemokines, ROS participate in a cascade of events leading to muscle regeneration and repair; on the contrary, the local persistence of ROS sustained by infiltrated neutrophils may cause further injury by oxidatively damaging differentiating myoblasts and myotubes thus delaying the *restitutio ad integrum*. Similarly, ROS generated during exercise promote mitochondriogenesis (a key factor in muscle differentiation) *via* peroxisome proliferator-activated-receptor-gamma-coactivator-1*α*-(PGC-1*α*) activated signal transduction pathway [3] but, at higher and persistent levels, they might target mitochondria and mitochondrial DNA (mtDNA) turning into blockers of myogenic differentiation [4, 5]. Other examples of such diverging capacities—which strengthen the generally accepted notion that ROS act in a hormetic fashion—will be discussed thereafter. The prevalence of each of the two actions, that is, beneficial or detrimental, depends on the coincidence of various intrinsic and extrinsic factors among which the most prominent is the level and the duration of ROS targeting muscle cells; other variables are the source or the site of ROS generation, the antioxidant status of target cells, and their DNA repair capacity. The differentiation stage of muscle cells (satellite cell, differentiating myoblast or mature myotube) is also capable of redirecting the cell through different signaling pathways and of further modulating the ensuing cell response. Today ROS are known to trigger and/or affect many signaling pathways relevant to skeletal muscle cells' homeostasis and adaptation: here we will illustrate some of the signaling pathways triggered/affected by ROS in muscle tissue and their physiopathological implications (see Figure 1 for a visual summary).

## 2. Generation of ROS in Skeletal Muscle Cells

Mitochondria are commonly considered as the predominant source of ROS in skeletal muscle cells [6, 7]. Increased mitochondrial ROS generation occurs during various and different situations, such as in the course of intense contractile activity [8] or in response to cytokines such as tumor necrosis factor-*α* (TNF-*α*) [9]. Early reports assumed that 2–5% of the total oxygen consumed by mitochondria may undergo one electron reduction with the generation of superoxide [10, 11]. More recent studies indicated that complexes I and III of the electron transport chain are the main sites of mitochondrial superoxide production [12, 13]. During exercise, it is assumed that the increased ROS generation in the course of contractile activity is due to the high oxygen consumption that takes place during increased mitochondrial activity. Indeed superoxide generation in skeletal muscle increases to about a 50- or 100-fold during aerobic contractions [14, 15].

However, recent evidence demonstrates that mitochondria may not be the prevalent source of ROS during exercise [8], and future studies are required to better elucidate the mitochondrial role in contraction-induced production of ROS in skeletal muscle. In 2002 St. Pierre and colleagues [16] reexamined the rate of mitochondrial ROS production and concluded that the total fraction of oxygen converted into superoxide was equal to 0.15%; this value is significantly lower than that (2–5%) estimated by other authors (see for example [17]). This lower rate of superoxide production takes account of the uncoupling proteins role (specifically UCP3 in skeletal muscle) as regulators of mitochondrial ROS production [18, 19] acting to prevent oxidative damage to mitochondria. In addition, growing evidence highlights that mitochondria produce more ROS during the basal state 4 of respiration as compared to state 3 (maximal ADP-stimulated respiration) [20–23]. Thus, since skeletal muscle mitochondria, during aerobic contractile activity, are predominantly in state 3, this limits their capacity of generating ROS during contractions [21–23]. 

Mitochondria are not the main and only source of ROS production in skeletal muscle during exercise. Indeed, other relevant sources of ROS production within muscle cells are nicotinamide adenine dinucleotide phosphate (NADPH) oxidases (NOXs) located within the sarcoplasmic reticulum, transverse tubules, and the sarcolemma [24, 25]. 

Phospholipase A2 (PLA2) is another known source of intracellular ROS production [17]. Arachidonic acid released from cell membranes by PLA2 is a substrate for ROS-generating enzyme systems such as the lipoxygenases [26]. Activation of PLA2 can stimulate NOXs [27] and increased PLA2 activity can promote ROS production in muscle mitochondria [28] and cytosol [29] and release ROS into the extracellular space [26]. Both calcium-dependent and -independent forms of PLA2 exist in skeletal muscle and both contribute to muscle ROS generation [17]. In particular, the calcium-independent isoforms are likely to be involved in cytosolic oxidant activity in skeletal muscle cells [29], whereas a 14-kDa calcium-dependent isoform located within mitochondria is reputed to stimulate mitochondrial ROS generation during contractile activity [30]. In this light it has been proposed [29] that the calcium-independent PLA2 is a major determinant of ROS activity under resting conditions, whereas during processes or stress elevating intracellular calcium concentration (contraction, inflammation, heat stress, etc.) the calcium-dependent PLA2 is activated to promote ROS production.

Finally, superoxide anion is known to be generated by xanthine oxidase (XO) in the cytosol of contracting rat skeletal muscles cells [31]. However, human skeletal muscles contain lower levels of XO than rat muscle cells, and the question whether XO plays an important role in superoxide production in human skeletal muscle is still open [31, 32]. More ROS-generating mechanisms may be operative at the same time, as in the case of prolonged muscle ischemia where mitochondrial and cytoplasmic (*via* XO) ROS production has been simultaneously scored [33].

ROS can be generated through the above mechanisms not only within muscle cells but also in their proximity. For instance, during inflammation (a pathophysiological state that substantially alters cellular oxidative/antioxidant homeostasis) infiltrated polymorphoneutrophils activate NOX producing ROS *via* the respiratory burst and many cytokines, which amplify in a feedforward cycle ROS production, are secreted within muscles [34]. For instance, during the early phase of muscle injury, inflammatory cytokines can bind to membrane receptors and activate specific ROS-generating enzymes, such as cyclooxygenase-2, NOX, and XO [35]. Endothelial cells from injured muscle are known to secrete TNF-*α*, interleukine (IL)-1, IL-6, and IL-8, providing a positive feedforward cycle [36]. Whereas a transient oxidative stress is necessary in inflamed muscle cells to exert an antiseptic function and to activate various signal transduction pathways relevant to the *restitutio ad integrum*, prolonged severe oxidative stress may imbalance intracellular antioxidant homeostasis and hence long-term muscle welfare.

The oxygen-centered species that mostly arises from the processes described so far is superoxide anion, but its significance in ROS signaling pathways seems to be limited to a role as a precursor signaling molecule. Indeed superoxide undergoes enzymatic or spontaneous dismutation, a process generating H_2_O_2_. H_2_O_2_ is a nonradical, is a weak oxidant with a relatively long half-life allowing its diffusion within cells and across cell membranes [37], reacts with many different cellular molecules, and activates a wide number of signaling pathways. These properties render H_2_O_2_ the most relevant ROS signaling molecule in cells [38]. In contrast, H_2_O_2_ undergoing Fenton chemistry in the presence of redox active free iron ions or other transition metals can give rise to hydroxyl radicals, which react immediately with any surrounding biomolecules exerting most of the deleterious effects associated with oxidative stress. In this light, iron homeostasis can be considered as a comodulator of ROS signaling and effects. In particular, since skeletal muscle contains 10 to 15% of body iron—mainly in myoglobin and mitochondria—it could be particularly sensitive to alterations of iron homeostasis: accordingly it has been recently reported that levels of muscle nonheme iron and the iron transport protein, transferrin were elevated in senescence, suggesting that iron load is a significant component of sarcopenia [39].

## 3. Antioxidants and Modulation of Muscle Cells' Sensitivity to ROS 

As it will be discussed throughout this review, the net effect of ROS on cells' signaling pathways and fate depends also on the cellular antioxidant capacity. The antioxidant network consists of enzymes, such as catalase, glutathione peroxidase (GPx), thioredoxin reductases (TRxs), superoxide dismutases (SODs), and soluble antioxidants such as glutathione (GSH) and vitamin E. Depending on its efficacy, the antioxidant cellular network plays a primary role in maintaining ROS below a physiologically compatible threshold level, thus allowing ROS to serve, theoretically, as signaling molecules and avoiding them to exert direct toxic effects. Many antioxidant enzymes are known to be induced in response to increased ROS generation. The increased ROS flux occurring in the course of strenuous exercise, through redox-sensitive mechanisms, induces the expression of *γ*-glutamylcysteinyl synthetase, the rate-limiting enzyme of GSH synthesis, of GPx, and of MnSOD [40]. Nuclear Factor KappaB (NF-*κ*B), activator protein 1 (AP-1), and mitogen-activated protein kinases (MAPKs) have been identified as the major signaling pathways that can be activated by exercise-derived ROS and directly involved in the induction of the above antioxidant enzymes [40]. For instance, in the signaling path of MnSOD gene expression, NF-*κ*B and AP-1 play an important role [41]: both NF-*κ*B and AP-1 binding sites are present in the promoter of the mammalian MnSOD gene, and ROS have been shown to activate their binding. 

The above responses, whose extent might be genetically predetermined, represent a fundamental adaptive countermeasure to conditions potentially resulting in frank oxidative stress. A specific modulator of ROS activity, working in an “antioxidant-like” fashion in ROS-mediated autophagy and apoptosis (see also “*ROS mediate autophagy and apoptosis*”), is the p53-inducible glycolysis and apoptosis regulator (TIGAR), a p53-target gene. Indeed, TIGAR can reduce ROS levels in response to nutrient starvation or metabolic stress, thus inhibiting autophagy and apoptosis independently of mammalian target of rapamycin (mTOR) or p53 modulation [42]. The TIGAR protein functions as a fructose-2,6-bisphosphatase and contributes to the regulation of intracellular ROS levels by modulation of the glycolytic pathway [43]. By decreasing glycolytic rate and redirecting glycolytic intermediates to the oxidative branch of the pentose phosphate pathway, TIGAR causes an increase in NADPH production, which favours ROS scavenging through GPx/glutathione reductase and GSH cycle. In this manner, TIGAR lowers the sensitivity of cells to ROS-induced p53-dependent apoptosis [43]. 

As to the influence of physical exercise, it caused an activation of MAP kinases in gastrocnemius muscle from rats; this in turn activated the NF-*κ*B pathway and consequently the expression of SOD and adaptation to exercise through increased expression of endothelial and inducible nitric oxide synthase [40]. All these responses were silenced when ROS production was prevented by allopurinol [40]. Thus ROS act as signals in exercise because their scavenging prevents activation of important signaling pathways promoting useful adaptations in cells. Because these signals result in an upregulation of powerful antioxidant enzymes, exercise itself can be considered an antioxidant. 

Besides the established soluble cellular antioxidants, creatine (Cr) is emerging as a pleiotropic molecule capable of influencing muscle cell's trophism, differentiation, and sensitivity to ROS [44, 45]. Cr has a high tropism for skeletal muscles, where most of body Cr is stored and has been shown to exert direct and indirect antioxidant activity in proliferating and differentiating C2C12 myoblasts [44]. A recent article by Young et al. [46] showed that two TRxs situated in the mitochondria and cytoplasm, respectively, were increased in Cr-treated C2C12 myoblasts: peroxiredoxin-4, a type 2 peroxiredoxin, and thioredoxin-dependent peroxide reductase. 

As it will be discussed below, the 5′-adenosine monophosphate-activated protein kinase (AMPK) signaling is critical in regulating mitochondrial content and function in a PGC-1*α* -dependent pathway in different tissues and in response to various stimuli [47]; furthermore AMPK signaling is important in preventing the mitochondrial dysfunction/impairment increasing ROS leakage and accompanying sarcopenia, disuse muscle atrophy, and other degenerative muscle disorders, in such a way that it can be considered as an indirect antioxidant cellular setting. Ceddia and Sweeney [48] firstly demonstrated that Cr supplementation may improve cellular bioenergetics by activating AMPK to improve overall mitochondrial content and/or function. It is currently unknown whether Cr supplementation exerts similar AMPK effects in oxidatively injured muscle tissue. At this regard, we recently observed in either control or oxidatively challenged differentiating myoblasts that a 24 h Cr preloading [0.3 mM] is an adequate stimulus to activate the AMPK pathway (unpublished observation).

This observation, along with others showing that Cr also acts as a direct antioxidant [5, 44, 49, 50] and protects differentiating myoblasts from H_2_O_2_-dependent differentiative arrest [5], suggests that in oxidative stressing conditions Cr treatment might confer myoblasts an enhanced adaptive capacity resulting in increased mitochondrial functionality and biogenesis and reduction of oxidative damage during myogenesis. Thus, besides established antioxidants, Cr might represent a skeletal-muscle-directed endogenous molecule capable of exerting multiple, pleiotropic actions which collectively help counteracting excessive ROS pressure. Consistently, beneficial effects of Cr supplementation have been reported for a large number of muscular, neurological, and cardiovascular diseases as well as in sarcopenia and aging [44, 51–56]. On the contrary, although great attention has been—and is still—paid to the administration of established antioxidants including polyphenols and vitamins in order to reduce the potential risk of the sustained and persistent action of ROS on skeletal muscle, there is no clear consensus on the benefits of these supplements [40, 57–59]. Thus, exploiting the efficacy of “atypical” and pleiotropic antioxidants such as Cr deserves consideration.

The integrity of the antioxidant network is particularly important in aging. Indeed, it is well-established that aging is associated with increased free radical generation and the resulting oxidative damage accumulated in organisms are likely to be involved, at least at a concausal level, in the progression of numerous diseases [60, 61]. It has long been suspected that senescent skeletal muscle may progressively loose its ability to adapt to oxidative stress [62, 63]. However, at the present, there is no clear consensus about how and whether senescent skeletal muscle becomes more susceptible to ROS pressure. For example, although in skeletal muscle antioxidant enzyme activities are increased with old age [64, 65], protein and mRNA levels of CuZnSOD, MnSOD, and GPx were found to be decreased or unaltered in the aged muscle [66, 67]. More importantly, aged muscle exhibited reduced antioxidant adaptation compared to training young muscle [62]. The reduced ability to rapidly activate an antioxidant adaptation program may render the senescent muscle more prone to oxidative damage. Notably, it has been hypothesized that the lack of adaptive capacity in aging muscle may depend on the impairment of signal transduction of antioxidant gene expression in response to oxidative stress [68]. At this regard, as discussed above, NF-*κ*B and AP-1 are known to play an important role in MnSOD gene expression [41]. The decreased binding of these nuclear factors, despite increased ROS generation found in aged muscles, would suggest that aging slows down molecular signaling of antioxidant gene expression. Thus aging seems to decrease the ability of aged muscle to express at least MnSOD as demonstrated by lower nuclear protein binding, mRNA levels, and unaltered enzyme protein [62]. The observed increase in MnSOD activity in the same setting might depend on a posttranslational modification (activation) of the enzyme molecules in aged muscle. In contrast to MnSOD, CuZnSOD showed increased protein content and activity with age in type II muscle in the absence of mRNA changes [66]. On the whole, these data suggest that the widely reported increase in antioxidant enzyme activities in aging skeletal muscle do not depend on enhanced gene transcription, but can rather derive from translational and/or posttranslational mechanisms. Since aged skeletal muscles are affected by augmented levels of lipid peroxidation, protein oxidation, and DNA damage, these compensatory increases in antioxidant enzyme activity are ineffective in counteracting increased ROS generation.

## 4. ROS, Mitochondrial Biogenesis and Function

It is well known that oxidative signals affect mitochondrial biogenesis, morphology, and function in skeletal muscle cells [69–71]: again, the effect of ROS seems to be bifaceted and controversial. Indeed, ROS may be important either in eliciting pathological effects leading to mitochondrial dysfunction and cell death ([72, 73], see also below “*Mitochondrial ROS Mediate Autophagy and Apoptosis*”), or play physiological roles promoting positive responses in mitochondrial biogenesis and function. Mitochondrial biogenesis is dependent on the expression of the mitochondrial genome and the nuclear genes that encode mitochondrial proteins [74]. An important pathway triggered by ROS is that leading to the upregulation of the mitochondrial biogenesis master gene PGC-1*α*. The PGC-1*α* transcriptional coactivator is a major regulator of energy metabolism [75]. It controls many aspects of oxidative metabolism, including mitochondrial adaptations, insulin-sensitizing *via* the upregulation of selected genes involved in fatty acid *β*-oxidation, glucose transport, and oxidative phosphorilation [76–79]. The mitochondrial biogenesis signaling activated by PGC-1 members family involves the transcription factors that regulate expression of nuclear genes such as nuclear respiratory factor (NRF) 1/2 and estrogen-related receptor-*α* (ERR-*α*). These three latter genes control the expression of nuclear genes encoding mitochondrial proteins and induce expression of mitochondrial transcription factor A (T-fam), which regulates mtDNA replication and transcription, thus activating the coordinated expression of mitochondrial proteins [80, 81].

Several signaling kinases have been involved in mediating PGC-1*α* transcriptional activation in response to a variety of stimuli among which the most important are calcium/calmodulin-dependent protein kinase (CaMK) type IV [82], AMPK [83], and p38 mitogen-activated protein kinase [84]. Their activation induces the PGC-1*α* promoter transcriptional regulation [69]. Recently, it has been demonstrated that mitochondrial biogenesis in skeletal muscle is controlled, at least in part, by a redox-sensitive mechanism and that physical exercise, increasing the ROS production over the physiological level, stimulates the muscle PGC-1*α*/NRF-1/T-fam signaling [85]. Irrcher and colleagues have evaluated the link between ROS levels and PGC-1*α* gene expression [69] in C2C12 cells. They found that endogenously produced ROS, at least within skeletal muscle cells, are important for the maintenance of PGC-1*α* expression levels within a normal physiological range. Indeed, quenching basal endogenous ROS with N-acetylcysteine (NAC) results in reduced PGC-1*α* mRNA expression, an effect which is unrelated to any inhibition of PGC-1*α* promoter activity, but probably dependent on the enhanced instability of PGC-1*α* mRNA occurring in a low ROS environment. On the contrary, increasing ROS levels with exogenous H_2_O_2_ augments PGC-1*α* transcription indirectly *via* the AMPK activation caused by the oxidatively-induced ATP depletion. This stimulates the binding of USF-1 to an Ebox within the PGC-1*α* promoter, increases transcription and results in the induction of PGC-1*α* mRNA expression, whose stability would also be restored in a more ROS-rich environment. The interplay of PGC-1*α* and ROS is further strengthened by the fact that, besides being a key modulator of mitochondrial biogenesis, it is important in regulating the expression level of protective enzymes acting against ROS generation and damage [86]. Indeed, experiments with either genetic knockouts (KOs) or using RNA interference for PGC1*α* show that the ability of ROS to induce a ROS-scavenging program depends largely on PGC-1*α* activity [86]. This response includes genes encoding for antioxidant enzymes localized either within mitochondria (MnSOD) or cytosol (catalase and GPxs). Indeed, cells lacking PGC-1*α* are more susceptible to the toxicity induced by oxidative stress caused by H_2_O_2_ [86]. These latter effects of PGC-1*α* are likely to represent a compensatory response where it plays a central role in the adaptation of cellular energy metabolism, mitochondrial biogenesis and antioxidant capacity in response to oxidative challenge. At this regard and extending previous research from our group [5], we have recently addressed the problem of the role of PGC-1*α* in C2C12 myoblasts subjected to oxidative stress during the early stages of differentiation. In particular, we examined the effect of a mildly toxic concentration of exogenously added H_2_O_2_ [0.3 mM] on the regulation of PGC-1*α* expression and its relationship with AMPK activation (unpublished observations). According to Kang and Irrcher [69, 85], we found that 1 h treatment with H_2_O_2_ markedly increased PGC-1*α* mRNA expression. It is of worth that, concurrently, we also found an increased phosphorylation of AMPK as compared to untreated cells, suggesting that oxidative stress induces PGC-1*α* through the AMPK signaling pathway. However, despite the fact that challenged C2C12 myoblasts rapidly activate a defense-oriented signaling cascade, they displayed a 30–40% reduction of their viability as well as a survivors' reduced differentiative efficiency during the post-challenge incubation stage (up to 7 days of culture). This observation would imply that, besides probably being an obligatory and physiological response to ROS, activation of AMPK and of PGC-1*α* may not be sufficient to afford a complete protection to cells against an overwhelming oxidative stress. Accumulating or excessive oxidative stress is known to be detrimental for mitochondria: for instance mtDNA represents a critical target for oxidative damage [49]. Indeed, mtDNA mutations are known as being an etiological factor in oxidative stress-related disorders including cardiovascular diseases and inherited or acquired neurodegenerative disorders, mitochondrial myopathies, and the normal aging process.

## 5. ROS Mediate Autophagy and Apoptosis

ROS may trigger either autophagy or apoptosis: whether these two pathways will be activated depends on the cell context and on the availability of specific modulators of ROS activity [87]. Autophagy is one of the cellular defense mechanisms activated in response to an excessive ROS production. Indeed, ROS act as signaling molecules in the early events of autophagy induction [87]. Phosphoinositide 3 kinase (PI3K) is known to mediate, at least in part, ROS effects. If the prosurvival effort fails, ROS induce cell death which may involve either the autophagic or the apoptotic pathway, or both [72, 88].

ROS signaling pathways play an important role in the induction of autophagy under physio- and pathological conditions. In healthy cells, autophagy is routinely involved in organelles and proteins turnover as well as in cellular energetic balance [89]. One of its strongest and better-characterized stimuli is starvation, where mitochondrial ROS production is enhanced and autophagy increased [87]. Increased ROS generation in the mitochondria under starvation is known to depend, at least in part, by class III PI3K: this event is essential for the induction of autophagy [90]. Indeed, upon starvation, ROS, and in particular H_2_O_2_, oxidize and inhibit Atg4, a protease responsible for microtubule-associated protein (MAP) light chain 3 (LC3) delipidation, that is, a condition resulting in the stabilization of the lipidated forms of LC3 and promoting the autophagosome maturation [87]. Notably, the same authors reported that addition of antioxidants inhibits these effects, preventing autophagosome biogenesis [91].

Thus, autophagy induced by starvation, where ROS participate in a feedforward manner, plays a prosurvival role since it contributes to the mobilization and reutilization of diverse cellular energy stores [89].

In a different direction, it is also known that when autophagy is prolonged, it could lead to cell death independently from apoptosis [92]. Indeed in nonmuscle tissues and in specific pathological conditions, ROS-induced autophagy was often linked to cell demise and death. As to skeletal muscle, ROS have been implicated in the induction of autophagy in muscle atrophy, disuse, and aging [72, 93]. Important new evidence on the wasting effect induced by increased oxidative stress on muscle phenotype was obtained by targeting a mutant SOD variant found in human amyotrophic lateral sclerosis myopathy [93, 94]. Indeed, these authors created a mouse model with a G93A mutation of SOD1 restricted to skeletal muscle [93]: accumulation of ROS in the muscles of these mice induced progressive atrophy associated with increased autophagy and forkhead transcription factors O (FoxO3) expression, a transcription factor which controls the transcription of autophagy-related genes and is required for the induction of autophagy through the lysosomal pathway in skeletal muscle in the absence of AKT repression [95–97]. In addition, NF-*κ*B signaling has been proposed as an alternative pathway linked to ROS-mediated skeletal muscle atrophy [98]: indeed NF-*κ*B was found to induce muscle atrophy and wasting *via* the lysosomal enzyme cathepsin L [93, 99] upregulation. Since cathepsin L is typically upregulated by FoxO3, it might be speculated that ROS-induced NF-*κ*B converges on the FoxO3 autophagic pathway.

Increasing evidence suggests that autophagy of mitochondria is a selective and defense-oriented response against ROS, mitochondrial dysfunction and the accumulation of somatic mutations of mtDNA with aging [72, 100]. For this reason it has been recently proposed the term “mitophagy” to emphasize the nonrandom nature of this process [100]. Damaged mitochondria are removed by mitophagy by Binp3, a BH3 proapoptotic member of the Bcl-2 family and fis 1, a pro-fission mitochondrial protein that induces mitochondrial fragmentation and enhances the extent of mitophagy. Notably, inhibition/alteration of mitophagy can contribute to myofiber degeneration and weakness in muscle disorders characterized by accumulation of abnormal mitochondria and inclusions [101, 102]. 

ROS may have various and important roles in apoptotic cell death: direct actions such as oxidation of cellular proteins and lipids, damage of nucleic acids and functional alteration of organelles; ROS may also modulate cell death processes affecting various signaling cascades [103]. Indeed, ROS participate in early and late steps of the regulation of apoptosis, affecting different apoptotic signaling cascades in both intrinsic or extrinsic pathways. 

The extrinsic path, which involves stimulation of receptor-mediated apoptotic pathways, can be initiated by ligand-induced (e.g., TNF*α* and Fas-L and TNF-related apoptosis-inducing ligand, TRAIL) binding, which promotes the activation of caspase-3 and subsequent degradation of genomic DNA [103]. Recent evidence suggests possible direct roles for ROS in mediating death receptors activation and subsequent induction of apoptosis [104]. Indeed, apoptotic signaling is induced by NOX-derived ROS at the plasma membrane level, which lead to lipid raft formation and death receptor clustering activation [104]. The physiological relevance and significance of ROS-dependent receptor-mediated apoptosis as compared to the classical receptor/ligand-induced apoptotic signaling is, at present, incompletely understood and warrants further investigation. 

ROS may act as intracellular intermediates directly dysregulating the sarcoplasmic reticulum Ca^++^ flux and handling, which results in caspase-7 and calpain activation. Furthermore, ROS may cause mitochondrial swelling and fragmentation, and/or alter the conformation of the mitochondrial permeability transition pores (MPTPs), thus facilitating their opening and the release of proapoptotic proteins such as cytochrome c (Cyt C). Independently of caspase activity, apoptosis may follow the intrinsic path, where ROS may directly cause the release of mitochondrial endonuclease G (Endo G), and/or of apoptosis inducing factor (AIF), which is capable of promoting DNA fragmentation in skeletal muscle myonuclei [105].

Another protein coupled with ROS-induced apoptosis is the voltage-dependent anion selective channel protein 1 (VDAC1). This transmembrane protein has been defined a ROS sensor [106] that triggers opening of the MPTP complex under conditions of oxidative stress. Indeed VDAC1 is the main channel within the mitochondrial outer membrane and upon ROS accumulation exhibits an increased conductance associated with MPTP opening and dissipation of ΔΨ, thus favouring the efflux of apoptotic proteins located in the intermembrane space and finally cell death [107]. Notably, the pro- and antiapoptotic Bcl2-family proteins are released *via* VDAC1 action and ROS may further affect these responses as they are known, in nonmuscle cells, to down-regulate the endogenous levels of the antiapoptotic protein Bcl-2 [108]. The mechanism through which Bcl-2 levels are affected by ROS has been studied by Azad et al. in nonmuscle cell types and seems to depend on superoxide anion-related degradation of Bcl-2 protein through the ubiquitin-proteasomal pathway [109]. 

Furthermore, under oxidative stressing conditions, ROS activate a signaling cascade involving the protein kinase C (PKC) b-dependent phosphorylation of the Shc adaptor protein p66shc and its translocation to the mitochondrial matrix. In particular, the mitochondrially translocated fraction of p66shc behaves as redox enzyme that utilizes reducing equivalents derived from the mitochondrial electron transport chain to produce H_2_O_2_ in the intermembrane space, an event which is known to trigger apoptosis [110, 111]. 

The accumulation of ROS within the mitochondrial matrix, as well as their capacity of triggering apoptosis, is counteracted/regulated by mitochondrial antioxidant enzymes, namely, phospholipids hydroperoxide glutathione peroxidase, GPx, and Mn-SOD [3, 112].

Thus increased mitochondrial production of ROS is involved at multiple levels in promoting apoptosis in skeletal muscle cells, an event which participates in the aetiology and progression of numerous pathologies including sarcopenia and disuse muscle atrophy as well as in aging [71, 113].

Physical training and exercise are known to increase mitochondrial biogenesis and density as well as mitochondrial ROS production especially during repeated contractions [85]. Therefore and unless other determinants are considered, it might appear paradoxical that although a routine of regular exercise is associated with numerous health benefits, physical exercise might potentially promote oxidative stress and ROS-associated apoptosis of skeletal muscle cells [17]. Indeed, chronic contractile activity (CCA) and endurance training induce an adaptive response in skeletal muscle cells leading to increased mitochondrial biogenesis [114] and—theoretically—an obligatory increase in a number of proapoptotic mitochondrial proteins and byproducts such as ROS. However, as a matter of fact recent evidence indicates that mitochondria isolated from rat skeletal muscle subjected to CCA seem to acquire an antiapoptotic, rather than proapoptotic, behaviour [114]. The study also addressed the problem of the relative antiapoptotic role acquired by different mitochondrial subpopulations from CCA-trained muscles, namely, the intramyofibrillar (IMF) and the subsarcolemmal (SS) mitochondria. The release of both Cyt C and AIF caused by exogenous H_2_O_2_ from CCA-isolated IMF and SS mitochondria was decreased; CCA augmented the expression of antiapoptotic HSP70 and caspase recruitment domain protein in either SS or IMF and caused a decreased ROS generation in IMF mitochondria. On the contrary, states III and IV respiring SS mitochondria showed a modestly increased rate of ROS generation as well as an increased resilience of MPTP opening. It was then hypothesized that these effects might collectively reflect the overall reduced apoptogenic capacity acquired by mitochondria following CCA training of skeletal muscles and that, in particular, the slight increase of ROS generated by SS would contribute to the activation of redox-sensitive transcription factors promoting muscle fiber plasticity and adaptation, rather than to function as proapoptotic triggers. Again, such a scenario is indicative of the diverging effects that ROS may assume depending on specific situations of cells' life, rather than on their net concentration and site of generation. 

## 6. ROS Signaling and Myogenic Differentiation

Increasing evidence indicates that ROS are capable of affecting—mostly reducing—the efficiency of myogenic differentiation. The integrity/alteration of myogenic differentiation is central to many physiological and pathological processes. Successful differentiation of satellite-derived myoblasts into functioning and integrated myotubes is a fundamental prerequisite for muscle regeneration, a repair process which is of primary importance in maintaining muscle function [115]. Notably, oxidative stress is known to play a concausal and detrimental role in a variety of multifactorial muscular pathologies characterized by proliferation/differentiation imbalance such as Duchenne dystrophy [116], myotonic dystrophy [117], sarcopenia [118], and cachexia [119].

The role of ROS in this context has been extensively documented. Ardite et al. [120] showed that ROS induced a strong depletion of the intracellular GSH pool: notably depletion of GSH causes further intracellular accumulation of ROS which favors NF-*κ*B activation, thus contributing to the lower expression of MyoD and impaired myogenesis (see below). 

According to Ardite et al. [120], data from our group [5] indicate that a mildly toxic H_2_O_2_ treatment during the early stages of C2C12 myoblast differentiation results in GSH depletion and strongly impairs the differentiative outcome. This effect is unlikely to be a mere result of ROS-induced cell demise: indeed, the cells surviving H_2_O_2_, although exhibiting a partial and late recovery of protein synthesis and of viability, were unable to continue and execute the differentiative task. These cells also displayed a strong and long-lasting reduction of the mRNA levels of MyoD, which is involved in early stem cell commitment, and of myogenin and MRF4, both recruited at later differentiation times [121, 122]. Whether the transcription of these muscle regulatory factors (MRFs) is a result of a specific signaling promoted by ROS or of a cell suffering is still to be understood. Under the same conditions depressing these MRFs, insulin-like growth factor 1 (IGF-1) which plays a pivotal role in controlling muscle growth [123], was inhibited to an even greater extent (see also “ROS and IGF-1 signaling”). Interestingly, H_2_O_2_-injured cells showed signs of extensive mitochondrial degeneration (swelling and disruption) and lower mitochondrial density, suggesting that these organelles are specifically targeted by—or particularly sensitive to—exogenous ROS. Loss of mitochondria is a clearly detrimental event in a process typically requiring active mitochondriogenesis such as muscle differentiation [4, 124]. 

ROS generated by the inflammatory cytokine TNF*α* are known to inhibit myogenesis, and this effect is widely attributed to oxidative activation of NF-*κ*B and subsequent gene expression [125–127]. However, the effect of TNF*α* is likely to be more complex since Langen et al. [9] showed that TNF*α* causes loss of myogenic capacity of C2C12 cells *via* NF-*κ*B-dependent and-independent and oxidative-sensitive and-insensitive pathways. In particular they hypothesized that an oxidative-sensitive, NF-*κ*B-independent mechanism might involve the blockage of the formation of functional catenin-adherin complexes proximate to the cell membrane [128]. Potentially, disruption of these complexes and the resulting alteration of cell-matrix and cell-cell interactions, might be responsible for the inhibition of myotube formation independently of NF-*κ*B. 

The redox regulation of the NF-*κ*B family of transcriptional activators plays a central role in differentiation, adaptation, and death of muscle cells. This role is extremely complex: indeed the effects promoted by NF-*κ*B are sometimes contradicting. As an example, although ROS can directly stimulate NF-*κ*B, oxidized NF-*κ*B has a diminished DNA-binding activity [17]. NF-*κ*B has been mostly associated with a negative regulation of skeletal muscle differentiation [119, 129, 130]. NF-*κ*B is constitutively active in proliferating myoblasts and can inhibit myogenesis by promoting a mitogenic activity *via* cyclin D1 or by inhibiting the synthesis of MyoD, a muscle-specific helix-loop-helix transcription factor operating in muscle development and repair [131–133]. More recently, NF-*κ*B was shown to suppress myofibrillar gene expression through the regulation of the myogenic transcriptional repressor Yin Yiang 1 [134]. Moreover, treatment of primary myoblasts with the NF-*κ*B inhibitor curcumin stimulates myoblast fusion thereby enhancing myogenesis and repair [125]. In line with these *in vitro* findings, activation of the TNF*α* pathway by muscle gene transfer inhibits regeneration *in vivo*, while muscle-specific deletion of the heteromeric kinase complex IKK was recently described to promote secondary myogenesis in response to acute injury signals [126, 127]. Activation of NF-*κ*B downstream ROS formation is also capable of stimulating the activity of inducible nitric oxide synthase (iNOS), whose role in myogenic process is controversial [24]. Some authors found that iNOS activity suppresses muscle differentiation, whereas others reported that stimulation of iNOS *via* NF-*κ*B represents a positive and necessary stimulus for muscle differentiation, that iNOS activity paralleled myogenesis from the early to later stages in H9C cells and that ROS formed by NOX 2 were the basic trigger leading to iNOS stimulation *via* NF-*κ*B recruitment [24, 135, 136]. Blockage of this pathway, or inhibition of iNOS with specific inhibitors, led to differentiative arrest. Also, a recent article by Lee et al. [137] indicates that complex-I-derived superoxide anions, produced through reverse electron transport, were dismutated into H_2_O_2_ by MnSOD induced *via* NF-*κ*B activation and that H_2_O_2_ stimulated muscle differentiation as a signaling messenger. Thus the scenario arising from these results would indicate that ROS negatively or positively regulate muscle differentiation *via* the signaling pathways involving NF-*κ*B activation. 

Another evidence which lends support to the detrimental role of ROS in muscle differentiation comes from the studies on the role of p66Shc in skeletal muscle ischemic injury. p66Shc, along with its isoforms p46 and p52, constitutes the mammalian Shc adaptor protein group. The three isoforms share a common structure, but p66ShcA has the unique feature of an additional domain at the N terminus which contains a serine residue at position 36 (Ser-36) that is phosphorylated in response to several stimuli, including H_2_O_2_. Due to this feature p66 isoform regulates ROS metabolism and apoptosis [138, 139]; indeed, a fraction of p66ShcA is localized in the mitochondria where, as discussed above, it produces mitochondrial ROS as signaling molecules for apoptosis [110, 111]. Interestingly, both p66ShcA KO cells and mice display lower levels of intracellular ROS [139–141] and are less prone to apoptosis induced by an array of different stimuli. Also, p66Shc KO mice are resistant to ischemia-induced apoptosis and show decreased muscle damage in response to hind limb ischemia [142]. More recently, Zaccagnini et al. [143] unravelled the role of p66Shc and ROS in muscular damage and regeneration following acute hind limb ischemia in both WT and p66Shc KO mice. WT mice showed detectable levels of oxidative stress markers during the postischemic and regenerative stages; on the contrary, the same markers were undetectable in KO mice. More interestingly, although the initial ischemic damage was identical and no advantage in terms of muscle vascularization and perfusion was observed in KO mice, their regenerative capacity was significantly higher as compared to WT. Satellite cell populations were similar in both groups, but those from KO mice showed a higher proliferation rate at first and spontaneous differentiation when cultured under prodifferentiative conditions. Finally, p66Shc KO satellite cells were resistant to the myogenic inhibition induced by H_2_O_2_ acute challenge or hypoxia. The authors proposed different and possible explanations for the above effects. The first one involves the different availability of NO—whose promyogenic role has been discussed above—in KO mice: since active p66Shc generates superoxide anions, which consume available NO forming the toxic species peroxynitrite, p66Shc KO mice would benefit of higher NO availability and would not suffer of peroxynitrite toxicity, two effects favouring myogenesis and muscle regeneration. Another plausible mechanism involves the NAD^+^-dependent histone deacetylase Sir2. Sir2 deacetylase activity is dependent on the fluctuation of cytosolic NAD^+^/NADH ratio, that is, the cellular redox state [144]. Under conditions of high ROS concentrations, NAD levels increase and promote Sir2 activation, which in turn inhibits MyoD-dependent transcription. p66Shc KO mice are characterized by lower levels of ROS and, as a result, decreased Sir2 activity, that is a condition which affects MyoD functions to a lesser extent. Finally, since oxidative DNA damage may trigger a differentiation checkpoint and cause a reversible inhibition of myogenic differentiation targeting MyoD phosphorylation, such a checkpoint activation may be attenuated by p66ShcA deletion, which results in decreased intracellular ROS levels. 

With regard to prodifferentiative effects induced by ROS in this context, in addition to the already cited report by Lee et al. [137], it has been recently demonstrated that in a non skeletal-muscle cell, that is, vascular smooth muscle cells (VSMC), ROS increase their differentiation rate after quiescence through a p38 MAPK-dependent pathway [145]. Similarly, other studies focusing on ROS and muscle metabolism, differentiation, and growth unravelled some positive interactions with IGF-1 signaling (see below).

Again, the most likely explanation for these opposite effects is that cell fate may depend on the intracellular ROS type (i.e., which is the prevailing reactive species) and level. In fact, it is well known that ROS elicit a wide spectrum of cellular responses, depending on their intracellular level [146]. A low dose of ROS controls normal cellular signaling pathways while an intermediate dose results in either temporary or permanent growth arrest [147]. Obviously, a high dose of ROS causes cell death *via* either apoptotic or necrotic mechanisms [142].

## 7. ROS and IGF-1 Signaling 

Growing evidence suggests that oxidative stress is responsible, as a causal or a concausal factor, for the pathogenesis of many muscle diseases and muscle wasting [148, 149]. In muscle cells, IGF-1 is known to promote muscle welfare inducing muscle hypertrophy and stimulate muscle-cell proliferation, differentiation, and survival [123]. IGF-1 has also been found to contribute to oxidative balance and to mediate protective responses against iron-induced-lipid oxidative stress *in vivo* [150]. Accordingly, Yang and colleagues [151] demonstrated that IGF-1 displayed protective effects on muscle cells after oxidative stress: indeed, pretreatment with IGF-1 protected muscle cells from H_2_O_2_-induced cell death and enhanced their survival through promotion of the antiapoptotic protein Bcl2. The same authors showed that protection was *via* an IGF-1 subpathway: PI3K/Akt and ERK1/2 MAPK pathways [151]. 

IGF-1 is a peptide hormone with a complex post-trascriptional regulation, generating distinct isoforms, namely, IGF-1Ea, IGF-1Eb, and IGF-1Ec (this latter also known as mechano growth factor, MGF) [152]. Mouse models have provided insights into the tissue-specific functions and responses to ROS of the different IGF-1 isoforms [152–155]. For example, in murine models, the local muscle isoform of IGF-1 (mIGF-1, the orthologue of human MGF) has been shown not only to activate proliferation of myoblasts [156], but also to protect cardiomyocytes from oxidative stress *via* the Sirtuin 1 deacetylase activity [157]. 

As to physical activity, although its role in regulating the expression of specific IGF-1 isoforms has been widely studied, data in the literature regarding humans are often contradictory and are affected by many uncontrolled variables such as the lack of dietary control, heterogeneity of subjects, their physical fitness, differences in proposed physical exercise, and time course of sampling [158–160]. 

Similarly to other pathways, ROS may regulate either positively or negatively IGF-1 signaling [161]. Low levels of endogenous ROS—due to their reversible oxidative inhibition of protein tyrosine phosphatases (see also “ROS as multipurpose local regulators of muscle cell functions”)—induce the phosphorylation on specific tyrosine residues of insulin receptor (IR) and IR substrates (IRS) protein(s), thus facilitating the IGF-1 signaling. Indeed, the IR*β* chain contains multiple sites for the phosphorylation of tyrosine that are sensitive targets of ROS such as H_2_O_2_ [162]. By contrast, higher ROS levels inhibit IGF-1 signaling cascades and recent evidence implicates ROS as downregulators of IGF-1 signaling and inducers of insulin resistance and its pathological sequelae [162].

However, ROS may be also involved in the activation of “insulin-like” metabolic effects by activating other non-insulin-initiated signaling pathways: one of the most important examples is the stimulation of glucose transport in skeletal muscle during exercise [163, 164]. Skeletal muscle contraction stimulates, as well as insulin, glucose transport by up to 50-fold during maximal exercise in humans [165]. Adding exogenous ROS to skeletal muscle *in vitro* stimulates glucose transport [166] whereas NAC, a potent antioxidant, reduces contraction-mediated glucose uptake by about 50% [167]. This effect of NAC was associated with a similar degree of inhibition of contraction-induced activation of AMPK. This kinase is a fundamental signaling kinase which, besides being involved in mitochondrial biogenesis (see “*ROS signaling and myogenic differentiation*”), is also known to upregulate the glucose uptake in muscle under conditions of high AMP/ATP ratio, like hypoxia and muscle contraction, forming a non-insulin-dependent pathway to increase muscle glucose utilization [158, 168–172]. Thus the proposed role of ROS in mediating the stimulation of glucose transport is related to skeletal muscle contraction, that increases superoxide anion production *via* mitochondrial respiration. Superoxide anion is rapidly converted to H_2_O_2_ by SOD, resulting in direct activation of AMPK, Glucose transporter 4 (Glut4) translocation to the plasma membrane, and an increase in glucose transport [173]. Moreover, in muscle cells, NAC antagonized ROS-mediated increase in glucose uptake in response to contraction, but not to insulin. Activation of AMPK in aerobic-exercise-induced glucose uptake is paradigmatic of ROS participation in physiologically-oriented signaling pathways relevant to the homeostasis of the entire organism. 

It has also been demonstrated that ROS regulate IGF-1-induced myotube hypertrophy *in vitro*. It is well known that exercise-induced muscle hypertrophy mostly depends on the increased local production of IGF-1 *via* activation of the PI3K/Akt pathway [174, 175]; interestingly ROS, which are being overproduced during exercise, contribute in a feedforward manner to stimulate IGF1 net accumulation. 

Previous reports show that there are positive and negative interactions between ROS and IGF-1 synthesis in both skeletal and VSMCs [176–178]. Treating VSMCs with H_2_O_2_ or XO augments both IGF-1 mRNA and IGF-1 protein secreted into the cultured medium, indicating that ROS enhance the IGF-1 autocrine system in VSMCs [176]. By contrast, we and others [5, 178] found that toxicologically relevant concentrations of H_2_O_2_ negatively regulate the IGF-1 mRNA levels in differentiating C2C12 myoblasts. In our experience, oxidative insult significantly decreased IGF-1 mRNA expression levels [5]. Cr, notably, prevented its inhibition: moreover Cr is known to induce hypertrophy of differentiating myoblasts *via* IGF-1 pathway [123].

Taken collectively, these results suggest that—although ROS enhance IGF-1 signaling—there is a negative feedback regulation of IGF-1 mRNA levels occurring with mildly toxic ROS levels in C2C12 cells. Thus, ROS regulate IGF-1 action *via* a variety of mechanisms, and the effects are likely, again, to be cell type and dose dependent. 

Thus, ROS play a crucial role in the IGF-1 signaling regulation and its biological action in muscle cells. However, additional studies are necessary to better explain the physiological significance of these interactions in humans, with particular regard to the identification of the distinct actions on the IGF-1 propeptide isoforms.

## 8. ROS as Multipurpose Local Regulators of Muscle Cell Functions

Similarly to other noninflammatory cells, skeletal muscle cells produce transient fluxes of ROS in response to an array of diverse stimuli, such as intense contractile activity [179, 180], heat stress [181], short-term disuse atrophy [182], acute hypoxia [143, 183], acute osmotic stress [184], and stretch [185]. Furthermore, locally produced waves of ROS are also released by skeletal muscle in response to cell surface receptor activation *via* cytokines, hormones, growth factors [186–188], or nuclear receptor activation [189, 190]. Considering the large variety of different stimuli converging to ROS production along with their lack of chemical specificity, it is hard to formulate a unitary explanation of the physiological significance of ROS in the responses triggered by such divergent signals [188]. At this regard, data published by Wright et al. [191] prompted these authors to draw an attractive hypothesis which involves the regulation of the protein phosphatases (PPases) “tone” in muscle cells and tissue. PPases belong to two broad families, the protein tyrosine PPases (about 112 human proteins) and the serine/threonine PPases (about 31 proteins). These two families are divided into further subclasses according to their specificity (only tyrosine targets or tyrosine plus serine/threonine targets) or, with regard to the second family, the subclasses characterized by a Zn^2+^/Fe^2+^ complex at the catalytic site or by the Mn^2+^/Mg^2+^ dependence [191]. 

The redox sensitivity of the protein tyrosine PPases and its potential biological importance is well documented *in vitro* and in cell culture systems since the early 1990s [192, 193]. As to Ser/Thr PPases, their sensitivity to oxidants is more controversial: calcineurin is the first whose sensitivity to oxidants has been clearly identified [194–196]. 

Interestingly, Wright et al. [191] found that not only protein tyrosine PPases, but also Ser/Threo PPases are inhibited by exposure to ROS or ROS generating agents (namely, H_2_O_2_ and DMNQ, resp.). The relative sensitivity of different PPases to oxidation in the above scenario has not yet been addressed. The mechanism by which PPases are oxidized is likely to involve the vulnerability of their ubiquitous and conserved cysteine-based active site; more surprising and still unexplained is the observed inhibition of ser/threo PPases which—with the exception of calcineurin—are best known in literature as “relatively immune to oxidation”. 

Indeed, the same study by Wright et al. [191] shows that in muscle tissue even minimal, physiologically relevant concentrations of oxidants, lead to an overall inhibition of PPases' activity. Notably, the concentrations used neither affected contractile function nor resulted in clear oxidative stress. Consistently, the level of net phosphorylation of a wide range of functionally diverging proteins was correspondingly higher in treated muscle preparations. This latter data suggests that oxidants are capable of affecting a broad range of PPases. Interestingly the majority of kinases are equally sensitive to oxidants but, contrary to PPases, oxidants promote their activation. Since kinases operate sequentially as amplification chains, it is likely that the observed increase in the net protein phosphorylation level under low-oxidative stressing conditions is the result of a lower PPases activity along with an increased kinases activity. These two combined events promoted by ROS would obviously trigger and/or affect many different signaling pathways, contributing to orchestrate the final cellular responses. 

In summary, oxidants could function to regulate *in vivo* global “phosphatase and kinase tone” and thus influence the kinetics and amplification of many kinase signaling pathways. With respect to skeletal muscle, such a scenario would be of great biological and physiopathological relevance, since muscle cells typically and continually produce ROS fluxes of different duration, intensity, and localization, depending on either intrinsic and extrinsic variables. Notably, such a hypothesis fits well with the hormetic nature of ROS.

## 9. Conclusion

The picture arising from this review indicates that ROS activate and/or participate in many signaling pathways promoting complex and diverging effects in skeletal muscle cells, ranging from positive to detrimental. As an example, many studies have concluded that inactivity-induced ROS production in skeletal muscle contributes to disuse muscle atrophy [197, 198]. On the contrary, growing evidence also suggests that intracellular ROS production is a required signal for the normal remodelling that occurs in skeletal muscle in response to repeated bouts of endurance exercise [40, 199, 200]. How can the same trigger promote such opposite effects? Based upon current knowledge, it appears that the mode and the situation characterizing skeletal muscle cells exposure to ROS may account, at least in part, for this apparent paradox. Transiently increased, moderate levels of oxidative stress might represent a potentially health-promoting process, whereas its uncontrolled persistence and/or propagation might result in overwhelming cell damage thus turning into a pathological event: for instance, the role of ROS in inflammation fits well with this model. In addition, the complexity, the variety, the interplay, and the functionally diverging roles of the signaling pathways activated or modulated by ROS contribute to further complicate this scenario. Thus, a gradual and variable, rather than a sharp, boundary is likely to characterize the transition between the two types of ROS actions. Such a variable “greyscaling” of ROS effects may depend on extrinsic and intrinsic situations such as, at least, (i) the concentration of ROS, (ii) the type of reactive species involved, (iii) the persistence of ROS activity, (iv) the localization of ROS source, (v) the antioxidant capacity and the energy status of muscle cells, (vi) their ability to adapt to oxidative stress (which *in vivo* also depends on ageing and/or physical training), (vii) the differentiative status, for example, myoblasts versus integrated myotubes, (viii) the absence/presence of an inflammatory process, and (ix) the plasticity of the signaling pathways triggered/affected by ROS. The balance between these factors will ultimately determine which type of signal(s) and effect(s) will prevail within the cell. Again, the hormetic nature of ROS emerges as the key feature of these species in many tissues, including skeletal muscle. Careful titration of ROS levels within skeletal muscle cell may therefore lie at the cross between the initiation and progression of disease and cell death, the induction of mitochondrial biogenesis, repair, and more generally cellular metabolic health. Supplementation with exogenous antioxidants is being widely studied to attain and maintain an “ideal titration” of ROS within skeletal muscle: unfortunately, at the present, no clear indication of the benefits arising from supplemental antioxidant intake emerges from literature. This reflects the need for further studies aimed at clarifying how to regulate ROS levels to exploit their physiological effects and avoid their damages.

## Abbreviations

AMPK: Adenosine monophosphate-activated protein kinase
Bcl-2: B-cell lymphoma 2
FoxO: Forkhead box O
GLUT4: Glucose transporter type 4
MAPK: Mitogen-activated protein kinase
NF-*κ*B: Nuclear factor kappa B
PGC-1*α*: Peroxisome proliferator-activated receptor gamma coactivator 1 alpha
PPases: Phosphatases
ROS: Reactive oxygen species
SOD: Superoxide dismutase.
